# Chronic Wound Management in Romania: A Survey on Practices, Protocols, and PRP Efficacy

**DOI:** 10.3390/medicina61061085

**Published:** 2025-06-13

**Authors:** Stefania-Mihaela Riza, Andrei-Ludovic Porosnicu, Cristian-Sorin Hariga, Ruxandra-Diana Sinescu

**Affiliations:** 1 Department 11, Discipline Plastic and Reconstructive Surgery, Bucharest Clinical Emergency Hospital, University of Medicine and Pharmacy Carol Davila, 050474 Bucharest, Romania; stefania-mihaela.riza@drd.umfcd.ro (S.-M.R.); cristian.hariga@umfcd.ro (C.-S.H.); ruxandra.sinescu@umfcd.ro (R.-D.S.); 2 Department of Plastic Surgery and Reconstructive Microsurgery, Elias Emergency University Hospital, 011461 Bucharest, Romania; 3 Clinic of Plastic Surgery and Reconstructive Microsurgery, Emergency Clinical Hospital Bucharest, 050474 Bucharest, Romania

**Keywords:** chronic wounds, wound management, platelet-rich plasma (PRP), clinical practices, treatment protocols, survey study, Romania, multidisciplinary care

## Abstract

*Background and Objectives:* Chronic wounds pose a significant challenge to healthcare systems, requiring long-term management and multidisciplinary approaches. The absence of a standardized national therapeutic protocol in Romania has resulted in inconsistent clinical practices, which in turn affect treatment efficacy and patient outcomes. The implementation of structured guidelines and the integration of regenerative therapies could enhance the management of chronic wounds. *Materials and Methods:* This study employs a cross-sectional observational design to assess the current management strategies among physicians treating chronic wounds and to identify variations in treatment approaches. A 37-question questionnaire was distributed among plastic surgeons, general surgeons, vascular surgeons, and dermatologists via Google Forms. The data collection period spanned one month, resulting in a total of 240 responses from medical centers in Bucharest, Romania. *Results:* The study found that most physicians treated several cases per week (40.8%) of delayed healing wounds, with the most frequent types being bedsores (57.5%) and diabetic (58.3%) or venous ulcers (55%). Challenges in wound care included patient reluctance, financial constraints, and the psychological burden on caregivers. The most relevant decision factor for surgical treatment was wound stage (86.7%). Most physicians used an initial conservative approach in wound care (52.5%) or did not have a standard approach (44.2%). Around a quarter of respondents (25.8%) used PRP as a treatment method, considering it to have moderate effectiveness (51.6%). The most important factor for encouraging PRP usage was having the necessary equipment for the procedure (72.5%). The most frequently considered benefit in the case of implementing a treatment protocol was increased treatment efficacy (62.5%). Physicians were also highly interested in the use of a standardized treatment protocol (40%). Approximately 41.7% of the physicians expressed a very high interest in having a standardized diagnostic system for chronic wounds. *Conclusions:* This study highlights that wound care practices remain variable and that the decision-making process is often challenging. There is a moderate belief in the effectiveness of PRP, suggesting that logistical barriers need to be addressed to facilitate access to it. Therapeutic protocols were seen as key to improving care efficacy and consistency, therefore pressing the need for national strategies that support protocol development.

## 1. Introduction

Chronic wounds represent a significant healthcare burden worldwide, characterized by prolonged healing, high recurrence rates, and a substantial impact on patient quality of life [1]. These wounds, which include diabetic foot ulcers, pressure ulcers, and venous or arterial leg ulcers, often require long-term, resource-intensive management and are associated with increased morbidity and healthcare costs [2]. Their persistence is frequently influenced by both clinical and socioeconomic factors, including comorbid conditions, limited access to specialized care, and patient non-compliance [3].

Chronic wounds are characterized by a disruption in the normal healing process, with wounds frequently becoming arrested in the inflammatory phase [4]. According to established criteria, if a wound fails to progress despite appropriate standard care, and the surface area reduction is less than 50% after four weeks of treatment, it is unlikely to heal without a change in therapeutic strategy [5].

The management of chronic wounds remains complex and varies considerably among clinicians. While standard care typically emphasizes a structured approach involving regular debridement, appropriate dressings, infection control, and patient education, treatment practices often diverge based on physician experience, specialty, and available resources [6]. Conservative management, focused on non-invasive therapies and wound bed preparation, is widely employed, particularly in the early stages of treatment [7]. In contrast, surgical approaches such as operative debridement, skin grafting, or flap reconstruction are often reserved for refractory or advanced cases; however, in some instances, they are considered early when the clinical condition requires it [8].

Among the newer modalities explored in chronic wound care is platelet-rich plasma (PRP), an autologous biologic product rich in growth factors (some examples include the following: platelet-derived growth factor—PDGF, transforming growth factor-beta—TGF-β, vascular endothelial growth factor—VEGF, and epidermal growth factor—EGF) that may promote tissue regeneration and accelerate healing [9]. While emerging evidence suggests its potential benefit, PRP is not yet widely adopted in practice, primarily due to logistical constraints and limited access to equipment, particularly in public healthcare settings [10]. Despite this, there is growing interest in PRP among physicians, reflecting a willingness to integrate novel regenerative therapies into standard wound care protocols.

Although chronic wounds are closely associated with important comorbidities such as diabetes and obesity, their actual impact on public health remains underestimated. By treating wounds merely as complications of other diseases rather than as a standalone clinical and economic burden, the impact of chronic wounds as a significant public health challenge has been largely overshadowed, highlighting the urgent need for dedicated investment in wound science and interdisciplinary care strategies [11].

While international protocols for chronic wound management are available, their consistent application in Romania appears limited. This discrepancy likely stems not from a lack of guidelines themselves, but from insufficient integration into clinical training, institutional practices, and everyday workflows. Consequently, addressing this gap requires not only national coordination but also enhanced education and systemic support to ensure that existing standards are effectively implemented in practice.

Considering these challenges and variations in clinical practice, the present study aimed to evaluate current wound care strategies among physicians from different surgical specialties, assess attitudes toward conservative versus surgical interventions, and explore perceptions surrounding the use of platelet-rich plasma (PRP). Furthermore, the study examines the perceived need for national standardized treatment protocols and diagnostic tools, a step considered essential for enhancing consistency, improving care quality, and optimizing patient outcomes in chronic wound management.

## 2. Materials and Methods

This study employed a cross-sectional observational design to assess current chronic wound management strategies among physicians and identify variations in therapeutic approaches. The primary objective was to evaluate how different specialties approach chronic wound treatment and the factors influencing the choice between conservative and surgical methods.

Data were collected using a 37-item questionnaire, distributed via Google Forms. The questionnaire was designed by a group of experts in wound management, all of whom are plastic surgeons, and pre-tested on physicians for clarity and relevance. The questionnaire was developed to capture demographic data, clinical practices, diagnostic and treatment preferences, and attitudes toward wound care. It included a mix of multiple-choice, Likert-scale, and open-ended questions.

The survey targeted plastic surgeons, general surgeons, vascular surgeons, and dermatologists involved in the care of chronic wounds. Respondents were recruited from various medical centers in Bucharest, Romania. Participation was voluntary and anonymous. The data collection period spanned January to March 2025.

The inclusion criteria were as follows: (1) physicians actively involved in the diagnosis and treatment of chronic wounds, including specialists in plastic surgery, general surgery, vascular surgery, and dermatology; (2) physicians working in public healthcare institutions in Romania; and (3) physicians with at least one year of clinical experience.

The exclusion criteria included the following: (1) incomplete or duplicate responses, and (2) physicians from other specialties not directly involved in wound care.

Criteria of Investigation: To structure the analysis, the survey was designed to assess five key domains:

(1) Demographic data of the respondents.
(2) Initial treatment strategy preference.
(3) Criteria for surgical decision-making.
(4) The use of platelet-rich plasma (PRP).
(5) Physician interest in standardized national protocols and diagnostic systems.

These domains were selected to provide a multidimensional view of current practices and to identify gaps in protocol-driven wound care.

Respondents were categorized into three groups based on their self-reported initial therapeutic approach to chronic wound management:

- **Conservative treatment group**—physicians who prefer to start with non-surgical, medical management strategies;
- **Surgical treatment group**—those who opt for surgical intervention as the initial strategy;
- **Individualized treatment group**—physicians who individualize treatment decisions without a fixed initial strategy.

Most of the variables collected were qualitative. Qualitative variables were reported as absolute frequencies (counts) and percentages, and associations between groups were analyzed using Fisher’s Exact Test. Where significant differences were identified in contingency tables, Z-tests for two-proportion differences with Bonferroni correction were applied for post-hoc analysis. Quantitative variables were expressed as means with standard deviations (SDs) for normally distributed data or as medians with interquartile ranges (IQRs) for non-normally distributed data. Normality was assessed using the Shapiro–Wilk Test. Between-group comparisons for quantitative variables with non-parametric distribution were performed using the Kruskal–Wallis H Test, followed by Dunn–Bonferroni post-hoc tests to identify pairwise differences. All statistical analyses were conducted using IBM SPSS Statistics, version 25, and visual representations of the data were created using Microsoft Office Excel and Word 2024. A significance threshold of *p* < 0.05 was used throughout the analysis.

## 3. Results

### 3.1. Physician Demographics and Approaches to Chronic Wound Diagnosis and Treatment

The three physician groups—initial surgical, initial conservative, and individualized treatment group—demonstrated distinct demographic and clinical practice profiles as seen in Table 1.

The initial surgical group (n = 8) consisted entirely of female physicians, with a median age of 36 years (IQR: 30–47.2 years). Most were from vascular surgery and dermatology specialties. This group reported routine use of imaging (100%), tissue perfusion tests (50%), photographic documentation (100%), and ankle-brachial index (ABI) measurements (50%), with significant differences compared to other groups (*p* = 0.001 for both). All reported routine bacteriologic sampling (100%), and 50% perceived multidrug-resistant (MDR) infections as “very frequent” (*p* < 0.001).

The conservative group (n = 126) had a median age of 31 years (IQR: 28–35), a higher proportion of male respondents, and strong representation from plastic surgery residency programs. Imaging was used by 38.1%, tissue perfusion by 25.4%, and all reported using clinical examination. None of the respondents rated clinical evaluation instruments as “very low” in effectiveness (*p* = 0.004).

The individualized treatment group (n = 106) had a median age of 33 years (IQR: 30–41.7) and included a mix of plastic and general surgeons, among others. Imaging was used by 35.8% (*p* = 0.001), tissue perfusion by 13.2% (*p* = 0.008), and patient history by 92.5% (*p* = 0.037). This group had the lowest ratings for evaluation tool effectiveness, with 13.2% rating them as “very low.”

Regarding MDR infection management, antibiotic treatment guided by antibiogram was reported by 86.8% of the no-standard, 84.1% of the conservative, and 100% of the surgical respondents (*p* = 0.621). Isolation measures were used by 49.1%, 36.5%, and 75%, respectively (*p* = 0.031), and local antibiotic treatment by 84.9%, 65.1%, and 75%, respectively (*p* = 0.002). Skin biopsy use differed significantly across groups (*p* = 0.013). Routine biopsy use was reported by 25% in the surgical group, while 37.7% in the no-standard group reported never using biopsies. Most respondents in all groups cited a dermatologist’s recommendation as the reason for the biopsy.

### 3.2. Determinants of Surgical Decision-Making in Chronic Wound Management

In the conservative group, 75% of respondents rated patient clinical stability as a very important factor before surgery. A total of 57.1% indicated preventing complications and supporting recovery as criteria for skin grafting. A total of 75% of respondents in this group reported avoiding surgery in high-risk patients, with a statistically significant difference between groups (*p* = 0.010). In the surgical group, 100% selected a lack of improvement with conservative treatment as a decisive factor for surgery. A total of 75% rated good clinical condition as very important. For skin grafting, 100% cited wound stage and well-vascularized wound bed as criteria. A total of 50% considered adequate wound bed preparation and rapid wound closure. A total of 25% selected aesthetic or functional reconstruction goals. These comparative findings are detailed in Table 2.

### 3.3. Clinical Perspectives on PRP Use and Protocol-Based Management Strategies

PRP was used by 50% of respondents in the surgical group, 31.7% in the conservative group, and 17% in the individualized treatment group, with a statistically notable difference between them (*p* = 0.008). Perceptions of PRP effectiveness did not differ significantly across groups (*p* = 0.221), with most physicians rating it as moderately or highly effective. The most commonly cited factors for increasing PRP use, such as access to equipment, financial support, additional training, and more clinical evidence, did not show statistically significant variation between groups. Regarding protocol implementation, the perceived benefit of improving treatment efficacy ranged from 60.3% to 75% across groups, while fewer respondents cited benefits such as cost reduction or clinical uniformity.

Interest in national wound management guidelines was generally high, with each group rating their interest as high or very high, but with no significant differences observed (*p* = 0.281). Among the perceived benefits of having an implemented protocol, increasing treatment efficacy was the most frequently reported across all groups, with 64.2% in the individualized treatment group, 60.3% in the conservative group, and 75% in the surgical group (*p* = 0.126). These comparative findings are summarized in Table 3.

Interest in national wound management guidelines was generally high across all groups. The proportion of respondents indicating high or very high interest ranged from 60.2% to 75%, with no significant variation observed (*p* = 0.281).

### 3.4. Patient Engagement, Home Care Strategies, and Digital Monitoring in Chronic Wound Management

Healing time was the most commonly reported patient concern, cited by 58.5% of the individualized treatment group, 49.2% of the conservative group, and 50% of the surgical group (*p* = 0.039). Socioeconomic and educational status were reported as a barrier by 66% of the individualized group, 49.2% of the conservative group, and 100% of the surgical group (*p* = 0.001). Other barriers included lack of modern equipment (52.8%, 52.4%, and 50%), lack of treatment guidelines (39.6%, 50.8%, and 25%), limited financial resources (43.4%, 44.4%, and 75%), and treatment non-compliance (66%, 65.1%, and 75%), none of which differed significantly across groups.

Regarding the perceived effectiveness of home care, 32.1% of the individualized group, 27% of the conservative group, and 50% of the surgical group rated it as very effective (*p* = 0.065). Moderate effectiveness was reported by 49.1%, 50.8%, and 25%, respectively. Home care use frequency differed significantly (*p* = 0.037), with very low use reported by 18.9% (individualized group), 28.6% (conservative), and 50% (surgical).

For telemedicine, 7.5% of the individualized group, 12.7% of the conservative group, and 25% of the surgical group rated it as very helpful (*p* = 0.020). It was considered beneficial in 52.8%, 50.8%, and 50% of cases, respectively. No usage was reported by 35.8%, 34.9%, and 0%, while 3.8%, 1.6%, and 25% reported it as without utility. A detailed overview of these aspects is presented in Table 4.

## 4. Discussion

This study offers a comprehensive examination of how physicians approach the complex task of managing chronic wounds, revealing distinct patterns associated with their approach to treatment, including whether they tend to start with conservative measures, adopt a non-fixed approach, or opt for early surgical intervention. The findings highlight an essential interplay between physician specialty, experience, and practice choices.

### 4.1. Diagnostic Patterns and Surgical Decision-Making

Our data on diagnostic practices highlight that an initial surgical mindset is associated with more rigorous diagnostic workups. Physicians anticipating surgical intervention frequently seek comprehensive information, including imaging to assess vascular status or underlying osteomyelitis, perfusion indices such as the ankle-brachial index (ABI) to evaluate healing potential, and wound cultures to guide perioperative antibiotic therapy. This thoroughness supports surgical planning and risk mitigation. In contrast, conservative-first providers often rely more heavily on clinical signs, reserving advanced diagnostics for wounds that fail to progress. While not every chronic wound requires extensive investigation, specific assessments, such as evaluation of arterial supply in leg ulcers, are critical for identifying underlying ischemia. The relatively low use of ABI testing among conservative providers in our study suggests potential under-utilization of vascular assessment outside vascular specialties. These findings parallel observations by Guest et al. (2020), who reported that only 15% of patients with a leg or foot ulcer in the UK had a documented ABPI, despite compression therapy being prescribed in a substantial proportion of cases, sometimes without confirming arterial status [12]. The lack of routine vascular assessment risks missing cases of critical ischemia, potentially contributing to stalled healing and poorer outcomes. Similarly, the widespread use of wound photography among surgical-first respondents in our study, in contrast to approximately 46% usage in other groups, underscores a missed opportunity for improving objective documentation. Guest et al. also emphasized that inconsistent diagnostic recording hampers evidence-based management [12]. The routine adoption of basic diagnostic measures, such as perfusion testing and photographic wound tracking, even outside surgical pathways, could enhance the quality of initial evaluation and support better-informed clinical decisions across all care models.

**Figure 1:**
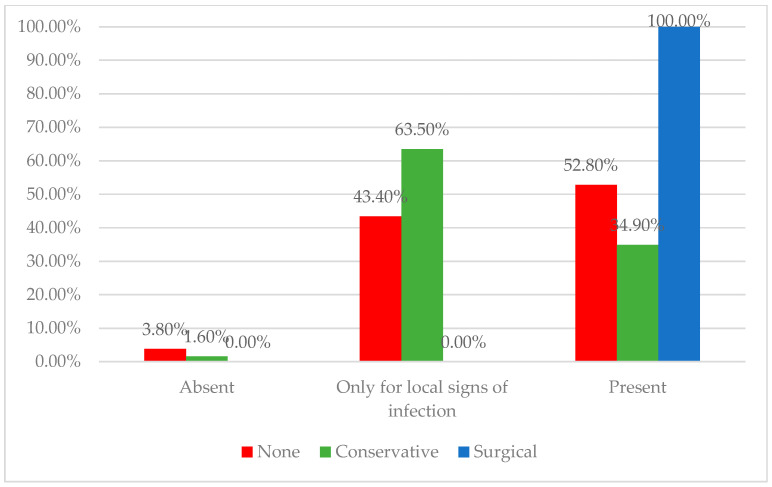
Distribution of physicians according to the usage of bacteriologic wound evaluations across treatment approach groups. The surgical-first group showed the highest rate of routine culture testing, whereas the conservative and individualized groups more often limited bacteriologic exams to cases with clinical signs of infection.

Regarding infection control, our findings align with the understanding that all chronic wounds should be assumed to harbor bacterial contamination [13], with bioburden levels critically influencing the healing potential [14]. Conservative providers, by culturing only when infection is clinically evident, reflect the principle that not all surface bacteria warrant treatment, as targeting non-pathogenic species may not aid healing [15]. In contrast, surgical providers, as illustrated in Figure 1, anticipate procedures and perform routine cultures to reduce bioburden and optimize wound bed preparation, especially given the role of biofilms in sustaining chronicity [16,17]. Both approaches emphasize the importance of striking a balance between reducing harmful bioburden and avoiding the overtreatment of colonizing flora, thereby supporting individualized wound management strategies.

Following diagnosis, providers must assess the healing ability of a wound, accepting that wound status may change over time. Chronic wounds are generally classified as healable, maintenance, or non-healable, with care strategies adapted accordingly. Healable wounds, which account for approximately two-thirds of community cases, have a sufficient blood supply and an identifiable cause, enabling progression toward closure. Maintenance wounds, accounting for approximately 25%, occur when adequate perfusion is present but healing is impaired due to patient non-adherence or systemic barriers. Non-healable wounds, comprising 5–10% of cases, result from irreversible causes such as inadequate perfusion or advanced disease, where management focuses on symptom control and quality of life [18]. In line with the Wound Healing Society guidelines, a change in therapy is recommended if no significant reduction in wound size is observed after four weeks of standard care, as data show that patients with less than 53% wound area reduction at four weeks have significantly lower rates of healing at 12 weeks [5,19]. Prolonged wound chronicity increases the risk of infection and complications, underscoring the need for timely reassessment and potential escalation of care [2]. Surgical intervention is typically reserved for cases where conservative measures fail or where healing is unlikely without operative management [5]. Our findings reflect this practice pattern: most physicians, 52.5%, favored initial conservative treatment, reserving surgical approaches for non-healing wounds where these measures fail or when the wound is unlikely to heal without surgery. The takeaway is that while surgery is a powerful tool, patient selection and pre-operative optimization remain paramount, and most physicians, including surgeons, respect that principle.

The observed differences in grafting practices reveal distinct clinical strategies among providers. Conservative-first physicians, primarily plastic surgeons (68.3%), tended to employ skin grafting as an active measure to expedite wound closure following appropriate wound bed preparation. In contrast, physicians in the no-standard group were more likely to delay grafting until ideal granulation was achieved. Both approaches are consistent with recognized principles of wound management. The factors commonly considered by physicians included local wound characteristics, such as a large wound surface area and a well-vascularized wound bed, as well as outcome-related goals, such as preventing complications and supporting recovery. This is consistent with reports showing that split-thickness skin grafts (STSGs) promote faster healing and lower recurrence rates compared to standard secondary intention management, particularly in diabetic foot ulcers, without significantly increasing surgical complications [8,20,21,22]. Consequently, the strategic use of STSGs may provide significant clinical advantages in selected patients [23,24,25].

### 4.2. Barriers to PRP Implementation

Recent studies have explored the use of stem cells and growth factors as new therapeutic strategies aimed at restoring the normal healing process [26,27,28]. Among these, platelet-rich plasma (PRP) has generated significant interest due to its high concentration of platelets, containing essential growth factors for tissue repair and regeneration, as well as its antibacterial properties [26]. PRP is defined as a plasma preparation with platelet concentrations 4–5 times higher than that of whole blood, typically ranging from 150 × 10^3^/dL to 400 × 10^3^/dL. The classical method for PRP preparation involves two centrifugation steps: first separating blood components into red cells, a buffy coat (rich in platelets and leukocytes), and platelet-poor plasma, followed by harvesting the concentrated platelets into a small plasma volume [29]. Although multiple studies have evaluated the efficacy of PRP in diabetic foot ulcer (DFU) treatment, results remain mixed. Several reports [26,30,31,32], including that by Yammine K et al., have demonstrated that PRP promotes wound healing, accelerates ulcer closure, and reduces the incidence of adverse events. However, other studies, such as that of Gupta A et al., reported no significant benefit of PRP in ulcer healing outcomes [33].

In addition to liquid PRP, platelet-rich gel (PRG) represents another autologous product increasingly explored in chronic wound care. PRG is typically prepared by activating PRP with calcium chloride and autologous serum, resulting in a semi-solid matrix that is easier to apply and localize on wound surfaces [9,34,35,36,37]. Its gel-like consistency offers practical advantages for outpatient and home care settings, particularly for irregular or exudative wounds. Despite limited clinical adoption in Romania, PRG may hold promise as a more user-friendly regenerative therapy in the future.

Despite growing scientific interest in platelet-rich plasma (PRP) therapy for chronic wound management, our findings indicate that its adoption among physicians remains limited. PRP was used by only 50% of surgical-first physicians, 31.7% of conservative-first physicians, and 17% of those using an individualized approach. This low to moderate uptake reflects a gap between the promising biological rationale for PRP, supported by experimental and some clinical studies, and its routine clinical application. Barriers to wider adoption likely include limited access to specialized equipment, lack of reimbursement or financial support, and the heterogeneity of existing evidence regarding clinical efficacy. Interestingly, despite the relatively low use of PRP, physician confidence in its effectiveness was moderate to high across all groups, suggesting that logistical and systemic factors, rather than skepticism about efficacy, are the primary obstacles to its widespread adoption. These findings underscore the need for broader infrastructural support and more straightforward clinical guidelines if PRP therapy is to be more widely integrated into standard wound care protocols.

### 4.3. Support for Protocols and National Guidelines

**Figure 2:**
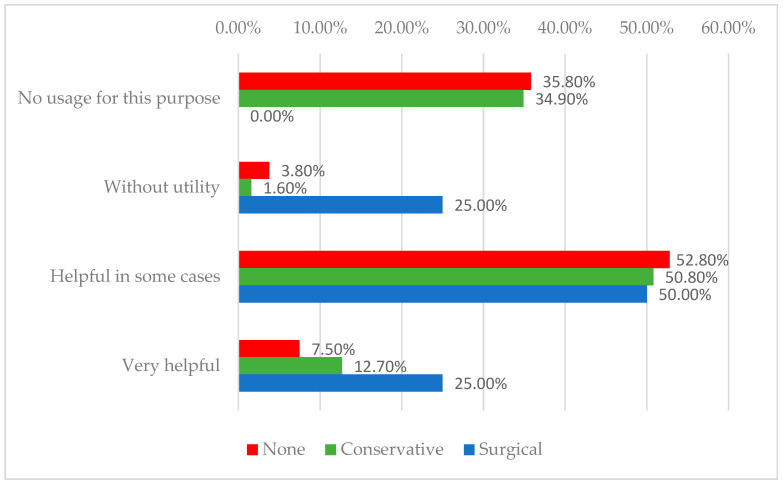
Distribution of physicians according to their interest in telemedicine monitoring and treatment approach.

Research consistently shows that engaging patients in care planning, involving them in decision-making, and providing continuous education on self-care and prevention are key strategies for improving patient concordance [38,39]. In this context, home care services and telemedicine play crucial roles in extending patient-centered care beyond the clinic, supporting education, monitoring wound progress, and promoting adherence [40]. By providing structured remote follow-up and reinforcing personalized treatment plans within the patient’s environment, these tools can enhance self-management, early detection of complications, and overall treatment success in chronic wound management [41]. In our case, telemedicine appeared less accepted among some practitioners, particularly surgeons, with 25% of the surgical group viewing it as lacking utility, likely due to concerns about the limitations of remote wound assessment. However, the general openness among other physicians suggests that broader adoption could occur with improved infrastructure and training. The frequent use of home care by a subset of non-standard providers may reflect practice settings with stronger home health programs or a higher proportion of patients who are immobile. Overall, access to home nursing and telemedicine remains inconsistent, despite recognition of their potential benefits. The distribution of physicians according to their interest in telemedicine monitoring, stratified by treatment approach, is illustrated in Figure 2.

Best practice guidance has little value if not implemented as Patton et al. suggested and as demonstrated by numerous examples where evidence-based medical advancements failed to translate into improved patient outcomes [42,43]. The widespread interest in standardized protocols and diagnostic systems among physicians represents a clear call to action for professional societies and healthcare institutions. Developing comprehensive guidelines that are accepted across specialties could help reduce unwarranted variability in wound management. A universal wound assessment tool, like the TNM staging system in oncology, would be particularly valuable in ensuring consistent communication and treatment planning. Although tools such as the PUSH tool for pressure ulcers and the SINBAD system for diabetic foot ulcers exist, their uptake remains inconsistent [44,45]. With nearly 75% of respondents expressing strong interest in standardized diagnostic systems, there is a clear opportunity for a concerted, multidisciplinary effort to design or promote a universal scoring system that stratifies wound severity and directs appropriate interventions. Similarly, the creation of adaptable treatment algorithms—incorporating decision branches based on clinical parameters such as perfusion (e.g., an ABI of less than 0.5 leading to a vascular consultation) or infection—could enhance the uniformity of care without sacrificing clinical flexibility. Physicians in this study demonstrated readiness to adopt such tools, which could support more consistent care pathways and potentially improve patient outcomes. At the same time, our findings underscore the importance of maintaining flexibility within standardized frameworks. While protocols provide essential structure, patient-centered care demands room for individualization, particularly in complex or atypical cases [46]. The individualized group exemplifies the necessity of tailoring decisions to unique clinical scenarios. Therefore, any future guidelines must strike a balance between standardization and clinical judgment, providing structured pathways while allowing for deviations when justified by patient-specific factors.

Compared to similar surveys conducted in countries like the UK, Germany, or the US, our findings reveal several specific differences for Romania: the lack of national protocols (NICE in the UK, the Wound Healing Society guidelines in the US, or the German S3-Clinical Practice Guideline), a significantly lower adoption rate of PRP (surveys in Europe report PRP use in chronic wounds in up to 50–70%, while in our survey, only 25.8% of Romanian physicians reported using PRP), and lower adherence to diagnostic tools like ABI testing or perfusion scans [47,48].

#### Limitations

This study has several limitations that should be acknowledged. First, the imbalance in group sizes may have influenced the findings, with specific results potentially driven by a smaller number of respondents, particularly in the surgical treatment group. As such, caution is warranted when generalizing these results to the broader physician population. Additionally, the final sample size of 240 respondents was based on pragmatic feasibility. A post-hoc power analysis indicated that the study retained sufficient statistical power (>80%) to detect moderate effect sizes. Another significant limitation is the potential for selection bias, as participation was voluntary and recruitment primarily targeted physicians working in urban centers and academic hospitals. This may have led to the underrepresentation of perspectives from rural settings or primary care providers, thereby limiting the generalizability of the findings across the entire healthcare system.

Additionally, although the survey captured key specialties involved in wound care, it did not include other essential provider perspectives, such as those of primary care physicians, podiatrists, or wound care nurses. As such, the findings may not fully represent the interdisciplinary nature of wound management. Furthermore, this study did not assess patient outcomes, making it impossible to directly link different practice patterns to healing rates, complication rates, or healthcare resource utilization. Future research should aim to explore these outcome implications, such as whether proactive surgical interventions improve healing times or whether prolonged conservative management delays necessary operative care. While our study provides valuable insight into current physician practices, longitudinal studies are needed to correlate specific management approaches with clinical outcomes.

It should also be noted that the gender imbalance among respondents may constitute a potential source of bias. However, we regard it as not directly relevant to the study’s objectives and likely reflective of the actual gender distribution within medical specialties in Romania.

## 5. Conclusions

This study underscores the considerable variability in chronic wound management practices among physicians, with many favoring either a conservative or individualized treatment approach. Surgeons, in particular, acknowledge the significant burden posed by chronic wounds and express a clear need for structured, multidisciplinary solutions.

Despite the relatively low adoption of platelet-rich plasma (PRP) therapy, there is moderate confidence in its clinical utility and a strong interest in its broader use. However, practical barriers—such as limited access to necessary equipment—remain a significant obstacle. Addressing these logistical challenges could facilitate the integration of PRP and other advanced therapies into routine care.

Notably, the findings reveal a shared recognition among clinicians of the urgent need for national protocols and standardized diagnostic tools. Such frameworks are crucial for enhancing treatment consistency, informing clinical decision-making, and ultimately improving patient outcomes. Given the additional challenges posed by patient non-compliance, socioeconomic constraints, and caregiver burden, a coordinated national strategy to support education, resource allocation, and protocol implementation is both timely and necessary.

## Figures and Tables

**Table 1:** Comparison of demographic characteristics, diagnostic approaches, and management strategies according to treatment approach groups (* Fisher’s Exact Test, ** Kruskal–Wallis H Test).

Parameter/Approach	Individualized Treatment Group (n = 106)	Conservative Treatment group (n = 126)	Surgical Treatment Group (n = 8)	*p*-Value
**Age (Median (IQR))**	33 (30–41.7)	31 (28–35)	36 (30–47.2)	0.003 **
**Gender (Female) (Nr., %)**	68 (64.2%)	66 (52.4%)	8 (100%)	0.007 *
Medical Specialty (Nr., %)				
Dermatology	12 (11.3%)	20 (15.9%)	4 (50%)	<0.001 *
General surgery	24 (22.6%)	14 (11.1%)	0 (0%)	
Vascular surgery	6 (5.7%)	6 (4.8%)	4 (50%)	
Plastic surgery	64 (60.4%)	86 (68.3%)	0 (0%)	
**Etiology of Chronic Wounds (Nr., %) (Total)**				
Arterial	28 (26.4%)	24 (19%)	6 (75%)	0.002 *
Pressure ulcer	70 (66%)	68 (54%)	0 (0%)	<0.001 *
Diabetic	64 (60.4%)	72 (57.1%)	4 (50%)	0.785 *
Autoimmune	8 (7.5%)	8 (6.3%)	2 (25%)	0.146 *
Post-traumatic	26 (24.5%)	36 (28.6%)	2 (25%)	0.859 *
Venous	54 (50.9%)	70 (55.6%)	8 (100%)	0.018 *
**Diagnostic Tools Used in Chronic Wound Assessment (Nr., %) {Total}**				
Clinical examination	106 (100%)	124 (98.4%)	8 (100%)	0.534 *
Laboratory tests	60 (56.6%)	62 (49.2%)	6 (75%)	0.253 *
Imaging studies	38 (35.8%)	48 (38.1%)	8 (100%)	0.001 *
Patient medical history	98 (92.5%)	104 (82.5%)	6 (75%)	0.037 *
Tissue perfusion assessment	14 (13.2%)	32 (25.4%)	4 (50%)	0.008 *
**Effectiveness of Clinical Wound Evaluation Instruments as Rated by Physicians (Nr., %)**				
Very low (not effective)	14 (13.2%)	2 (1.6%)	0 (0%)	0.004 *
Low	14 (13.2%)	22 (17.5%)	0 (0%)	
Moderate	38 (35.8%)	32 (25.4%)	4 (50%)	
High	24 (22.6%)	40 (31.7%)	2 (25%)	
Very high (highly effective)	16 (15.1%)	30 (23.8%)	2 (25%)	
**Clinical evaluation methods (Nr., %) (Total)**				
Objective examination	106 (100%)	124 (98.4%)	8 (100%)	0.534 *
Dimension measurements	80 (75.5%)	92 (73%)	6 (75%)	0.925 *
Standard photography	48 (45.3%)	60 (47.6%)	8 (100%)	0.008 *
ABI	4 (3.8%)	10 (7.9%)	4 (50%)	0.001 *
**Physician Practices Regarding Bacteriologic Wound Evaluation (Nr., %)**				
Not routinely performed	4 (3.8%)	2 (1.6%)	0 (0%)	<0.001 *
Only for local signs of infection	46 (43.4%)	80 (63.5%)	0 (0%)	
Routine—samples are taken from all chronic wounds regardless of infection signs	56 (52.8%)	44 (34.9%)	8 (100%)	
**Reported Frequency of Multidrug-Resistant (MDR) Infections (Nr., %)**				
Very low (almost never)	2 (1.9%)	8 (6.3%)	0 (0%)	<0.001 *
Low	18 (17%)	38 (30.2%)	0 (0%)	
Moderate	40 (37.7%)	50 (39.7%)	2 (25%)	
High	38 (35.6%)	26 (20.6%)	2 (25%)	
Very high (frequently)	8 (7.5%)	4 (3.2%)	4 (50%)	
**Management Strategies for Multidrug-Resistant (MDR) Infections (Nr., %) (Total)**				
Antibiotic treatment—antibiogram	92 (86.8%)	106 (84.1%)	8 (100%)	0.621 *
Patient isolation	52 (49.1%)	46 (36.5%)	6 (75%)	0.031 *
Local antibiotic treatment	90 (84.9%)	92 (65.1%)	6 (75%)	0.002 *
Regular wound monitoring	70 (66%)	76 (60.3%)	6 (75%)	0.546 *
**Frequency of Skin Biopsy Use in Clinical Practice (Nr., %)**				
Never use	40 (37.7%)	54 (42.9%)	0 (0%)	0.013 *
At dermatologist’s recommendation	60 (56.6%)	56 (44.4%)	6 (75%)	
Routinely	6 (5.7%)	16 (12.7%)	2 (25%)	
**Perceived Importance of Multidisciplinary Assessment (Nr., %)**				
Very low	0 (0%)	0 (0%)	0 (0%)	0.057 *
Low	4 (3.8%)	10 (7.9%)	0 (0%)	
Moderate	26 (24.5%)	16 (12.7%)	0 (0%)	
High	18 (17%)	22 (17.5%)	4 (50%)	
Very high	58 (54.7%)	78 (61.9%)	4 (50%)	
**Reported Use of VAC Therapy for Chronic Wounds (Nr., %)**				
Very low (rarely or never use VAC therapy)	8 (7.5%)	10 (7.9%)	0 (0%)	0.113 *
Low	26 (24.5%)	22 (17.5%)	0 (0%)	
Moderate	28 (26.4%)	36 (28.6%)	0 (0%)	
High	32 (30.2%)	42 (33.3%)	4 (50%)	
Very high (routinely use VAC therapy as a standard component of treatment)	12(11.3%)	16(12.7%)	4 (50%)	

**Table 2:** Comparison of determinants of surgical decision-making according to treatment approach groups (* Fisher’s Exact Test).

Parameter/Approach	Individualized Treatment Group (n = 106)	Conservative Treatment Group (n = 12)	Surgical Treatment Group (n = 8)	*p*-Value
**Factors Influencing the Decision to Pursue Surgical Treatment in Chronic Wound Care (Nr., %) (Total)**				
Relevant Medical History (e.g., diabetes, cardiovascular disease)	62 (58.5%)	60 (47.6%)	4 (50%)	0.231 *
Presence of Antibiotic-Resistant Infections	56 (52.8%)	70 (55.6%)	2 (25%)	0.246 *
Patient Age and General Clinical Condition	56 (52.8%)	70 (55.6%)	6 (75%)	0.478 *
Lack of Improvement with Conservative Treatment	76 (71.7%)	94 (74.6%)	8 (100%)	0.223 *
Treatment Cost Considerations	28 (26.4%)	18 (14.3%)	2 (25%)	0.048 *
Wound stage	98 (92.5%)	104 (82.5%)	6 (75%)	0.037 *
**Importance of Patient’s Clinical Status in Surgical Decision-Making (Nr., %)**				
Very low (“The patient’s clinical status does not influence the surgical decision”)	0 (0%)	0 (0%)	0 (0%)	0.409 *
Low	6 (5.7%)	6 (4.8%)	0 (0%)	
Moderate	24 (22.6%)	34 (27%)	0 (0%)	
High	24 (22.6%)	36 (28.6%)	2 (25%)	
Very high (“The patient’s clinical status decisively influences the surgical decision”)	52 (49.1%)	50 (39.7%)	6 (75%)	
**Avoidance of surgery in high-risk patients (Nr., %)**				
Very low (“Clinical status does not prevent me from performing surgery”)	10 (9.4%)	6 (4.8%)	0 (0%)	0.010 *
Low	22 (20.8%)	10 (7.9%)	0 (0%)	
Moderate	32 (30.2%)	38 (30.2%)	6 (75%)	
High	20 (18.9%)	34 (27%)	0 (0%)	
Very high (“Clinical status always prevents me from performing surgery)	22 (20.8%)	38 (30.2%)	2 (25%)	
**Factors Considered in Skin Graft Decision-Making (Nr., %) (Total)**				
Large Wound Surface Area	80 (75.5%)	100 (79.4%)	8 (100%)	0.293 *
Well-Vascularized Wound Bed	96 (90.6%)	96 (76.2%)	8 (100%)	0.007 *
Preventing Complications and Supporting Recovery	32 (30.2%)	72 (57.1%)	4 (50%)	<0.001 *
Adequate Wound Bed Preparation (e.g., debridement)	46 (43.4%)	50 (39.7%)	4 (50%)	0.739 *
Need for Accelerated Wound	44 (41.5%)	54 (42.9%)	4 (50%)	0.888 *
Closure Aesthetic or Functional Reconstruction Goals	34 (32.1%)	42 (33.3%)	2 (25%)	0.969 *

**Table 3:** Comparison of clinical perspectives on PRP use and protocol-based management strategies according to treatment approach groups (* Fisher’s Exact Test).

Parameter/Approach	Individualized Treatment Group (n = 106)	Conservative Treatment Group (n = 12)	Surgical Treatment Group (n = 8)	*p*-Value
**PRP Use Among Respondents (Nr., %)**	18 (17%)	40 (31.7%)	4 (50%)	0.008 *
**Physician Opinions on PRP Efficacy (Nr., %)**				
Without impact	2 (11.1%)	4 (10%)	2 (50%)	0.221 *
Moderate effectiveness	8 (44.4%)	22 (55%)	2 (50%)	
High effectiveness	8 (44.4%)	14 (35%)	0 (0%)	
**Clinician-Identified Drivers for Expanding PRP Use in Practice (Nr., %) (Total)**				
Financial accessibility	40 (37.7%)	42 (33.3%)	2 (25%)	0.723 *
Having necessary equipment	78 (73.6%)	92 (73%)	4 (50%)	0.376 *
Additional training	40 (37.7%)	38 (30.2%)	0 (0%)	0.056 *
More clinical evidence	44 (41.5%)	58 (46%)	6 (75%)	0.198 *
**Perceived Benefits of Having an Implemented Protocol (Nr., %)**				
Increasing treatment efficacy	68 (64.2%)	76 (60.3%)	6 (75%)	0.126 *
Reducing treatment costs	12 (11.3%)	4 (3.2%)	0 (0%)	
Reducing variability of clinical results	6 (5.7%)	14 (11.1%)	0 (0%)	
Uniformization of clinical treatment	20 (18.9%)	32 (25.4%)	2 (25%)	
**Level of Interest in National Wound Management Guidelines (Nr., %)**				
Very low	2 (1.9%)	4 (3.2%)	0 (0%)	0.281 *
Low	8 (7.5%)	8 (6.3%)	2 (25%)	
Moderate	26 (24.5%)	20 (15.9%)	0 (0%)	
High	36 (34%)	56 (44.4%)	4 (50%)	
Very high	34 (32.1%)	38 (30.2%)	2 (25%)	

**Table 4:** Comparison of patient engagement, home care strategies, and digital monitoring practices according to treatment approach groups (* Fisher’s Exact Test).

Parameter/Approach	Individualized Treatment Group (n = 106)	Conservative Treatment Group (n = 12)	Surgical Treatment Group (n = 8)	*p*-Value
**Common Patient Concerns (Nr., %)**				
Healing time	62 (58.5%)	62 (49.2%)	4 (50%)	0.039 *
Home care	32 (30.2%)	28 (22.2%)	2 (25%)	
Prevention	4 (3.8%)	16 (12.7%)	0 (0%)	
Infection detection	4 (3.8%)	6 (4.8%)	0 (0%)	
Alternative treatment	4 (3.8%)	14 (11.1%)	**2 (25%)**	
**Perceived Obstacles in Chronic Wound Treatment (Nr., %) (Total)**				
Lack of financial resources	46 (43.4%)	56 (44.4%)	6 (75%)	0.243 *
Socioeconomic and educational status	70 (66%)	62 (49.2%)	8 (100%)	0.001 *
Lack of modern equipment	56 (52.8%)	66 (52.4%)	4 (50%)	1.000 *
Lack of treatment guidelines	42 (39.6%)	64 (50.8%)	2 (25%)	0.116 *
Treatment non-compliance	70 (66%)	82 (65.1%)	6 (75%)	0.880 *
**Physician Assessment of Home Care Efficiency (Nr., %)**				
Unsure	8 (7.5%)	2 (1.6%)	0 (0%)	0.065 *
Ineffective	12 (11.3%)	26 (20.6%)	2 (25%)	
Moderately effective	52 (49.1%)	64 (50.8%)	2 (25%)	
Very effective	34 (32.1%)	34 (27%)	4 (50%)	
**How Frequently Home Care is Employed (Nr., %)**				
Very low (never)	20 (18.9%)	36 (28.6%)	4 (50%)	0.037 *
Low	24 (22.6%)	38 (30.2%)	2 (25%)	
Moderate	44 (41.5%)	42 (33.3%)	2 (25%)	
High	12 (11.3%)	10 (7.9%)	0 (0%)	
Very high (frequently)	6 (5.7%)	0 (0%)	0 (0%)	
**Perceived Usefulness of Telemedicine in Wound Monitoring (Nr., %)**				
No usage for this purpose	38 (35.8%)	44 (34.9%)	0 (0%)	0.020 *
Without utility	4 (3.8%)	2 (1.6%)	2 (25%)	
Helpful in some cases	56 (52.8%)	64 (50.8%)	4 (50%)	
Very helpful	8 (7.5%)	16 (12.7%)	2 (25%)	

## Data Availability

The original contributions presented in this study are included in the article. Further inquiries can be directed to the corresponding author.
